# AI and knowledge driven computation of rock mass characteristic parameters across engineering projects

**DOI:** 10.1038/s41598-026-56581-8

**Published:** 2026-06-09

**Authors:** Zewang Zheng, Yunpei Zhang, Quan Xu, Kezhong Wang, Changchun Ren, Hao Cai

**Affiliations:** 1 College of Civil Engineering, Zhejiang University of Technology, Hangzhou, 310014 Zhejiang China; 2 State Key Laboratory of Basin Water Cycle Simulation and Regulation, China Institute of Water Resources and Hydropower Research, Beijing, 100048 China; 3 YaJiang Clean Energy (Beijing) Development Technology Co., Ltd, Beijing, China; 4 Sinohydro Engineering Bureau 6 Co., Ltd, Shenyang, 110169 Liaoning China; 5 Key Laboratory of Urban Underground Engineering, Ministry of Education, Beijing Jiaotong University, Beijing, 100044 China

**Keywords:** Tunnel engineering, Characteristic parameters, CatBoost, Effective rock-breaking, Cross-project data utilization, Rock mass quality prediction, Energy science and technology, Engineering, Solid Earth sciences

## Abstract

With the continuous development of underground construction, represented by transportation tunnels, municipal utility tunnels and hydraulic tunnels, accurately perceiving the rock mass quality ahead of the tunnel boring machine (TBM) face has become critical for ensuring construction quality and improving construction efficiency. Over the past 5 years, predicting rock mass quality using machine learning and other artificial intelligence (AI) algorithms has gradually become a research hotspot. However, the raw TBM tunnelling data are massive and noisy, and key challenges remain, including how to reasonably select effective input features and how to cope with the limited data available from newly built projects. Although the academic community has proposed indices such as the TPI and FPI to quantify rock mass boreability and to incorporate them as input features, their application in practical engineering remains challenging. Owing to the complexity of tunnelling operating conditions and the variability in data quality, existing approaches exhibit notable limitations. In particular, physics-based “definition-based” methods typically require the processing of large volumes of high-frequency tunnelling data, and their computed results are highly sensitive to data fluctuations, resulting in poor stability. In contrast, “fitting-based” methods constructed on regression relationships, while showing reasonable effectiveness under single-project conditions, rely heavily on prior assumptions (e.g., penetration-based hypotheses of rock-breaking force) and are strongly influenced by fitting performance. As a result, these methods struggle to adapt to varying geological and engineering conditions, leading to limited generalizability and robustness in multi-project scenarios, and thus remain insufficient to support large-scale engineering applications. To address these issues, this study proposes an AI- and knowledge-driven method for computing rock mass characteristic parameters. Firstly, a rock-breaking data filtering method is developed based on the effective conversion of disc cutter energy. Engineering data from three TBM projects with different diameters and geological conditions are utilized, including the Yinchuo Water Diversion Project (YC), the Yinsong Water Diversion Project (YS), and the Huanbei Water Diversion Project (HB). Based on these datasets, this paper proposes an innovative methodology for identifying high-efficiency rock-breaking stages. Then, knowledge-driven rock mass characteristic parameters (*a*, *b*, and the torque penetration index, TPI) are computed as input features. Lastly, an AI-driven strategy is then adopted to determine the prediction model, whereby CatBoost is selected to develop the rock mass class predictor and is tested across different projects. The results indicate that the proposed characteristic parameters are strongly correlated with rock mass quality. The prediction accuracies in the YC, YS and HB projects reach 85.60%, 86.48% and 88.89%, respectively, outperforming conventional methods overall. The proposed method provides new technical support for cross-project data utilization and real-time prediction of rock mass quality, and has important implications for construction safety and efficiency in newly built tunnel projects.

## Introduction

 Since the beginning of China’s 14th Five-Year Plan, the development and utilization of underground space have continued to accelerate, and the scale of tunnel construction has expanded steadily. By the end of 2024, 424 new railway tunnels had been opened to traffic, with a total length of 738 km; 2521 railway tunnels were under construction and 5634 were in planning, with a total length of approximately 13,742 km^1^. TBMs (tunnel boring machines), owing to their advantages of safety, high efficiency and low labor intensity, have been widely adopted in tunnel construction^2^.In recent years, benefiting from the rapid development of computer and artificial intelligence (AI) technologies and the accumulation of TBM operational data^3^, research on smart TBM technologies has gained increasing momentum.

Among these, machine learning (ML) learns data-driven mappings between inputs and targets to enable recognition and prediction, and has therefore been widely adopted. In addition, the digital twin (DT) paradigm has emerged as another major research direction. DT constructs an “physical entity-virtual counterpart” mapping by integrating multi-source information, thereby enabling real-time analysis and decision support. For example, Latif et al.^4^ proposed a hybrid DT approach for TBM performance visualization, improving information representation and management efficiency during construction. Sharafat et al.^5^ further explored DT-driven optimization of underground construction schemes to support decision-making on design parameters. DT is oriented toward process management and decision support and thus exhibits strong development potential. However, continuous updating of twin information in practical tunneling projects typically relies on high-density sensing, reliable communication, and substantial computing and system-integration capabilities, which increases the difficulty of field deployment and long-term maintenance. By contrast, ML emphasizes learning “input–output” relationships from historical or streaming data. Common algorithms include random forest (RF), support vector machine (SVM), gradient boosting/boosting methods, and deep learning models for time-series data(e.g., LSTM/GRU). Owing to its relatively lightweight implementation and ease of rapid deployment, ML is selected in this study as the primary approach to develop and validate practical models for key tasks under modest on-site requirements.

Based on these methodological paradigms, current TBM intelligence research mainly focuses on two categories of tasks: (1) prediction/optimization of tunneling parameters and (2) identification of surrounding rock quality. Parameter optimization often involves multi-objective trade-offs among efficiency, energy consumption, cutter wear, vibration, and safety^6,7^. Similar attention has also been paid in shield tunnelling, where multivariable parameter prediction and combination models have been investigated to support performance assessment and construction control^8^. Meanwhile, studies on excavation-induced deformation analysis, nonlinear response prediction of adjacent pipelines, and jacking-force analysis in pipe-jacking construction indicate that underground construction performance is influenced not only by operational parameters, but also by excavation effects and tunnel–structure interaction^9,10^. Together, these studies provide a broader theoretical and application background for both parameter-driven performance assessment and tunnel–structure interaction analysis in underground engineering. However, the objective functions, constraints, and definitions of “optimality” vary substantially across projects, resulting in relatively inconsistent research boundaries. In contrast, surrounding rock quality identification/classification aims to characterize objective geological conditions; the task definition is clearer and more generalizable, and it also constitutes a key input prioritized by on-site operations. Therefore, this work investigates ML-based prediction of surrounding rock quality.

Although ML is advantageous in handling complex nonlinear data, its prediction quality and accuracy strongly depend on the rational selection of input features^11^. TBM equipment typically acquires on the order of hundreds of data channels. In existing research, this is often handled through feature engineering—that is, extracting a limited number of parameters that exhibit strong mapping relationships with the output variables to serve as model inputs. This approach reduces the dimensionality of the input vector while improving computational efficiency, and is widely recognized as one of the most important parts of ML^12–14^. In addition, ML prediction performance depends heavily on the scale and quality of the sample data. Data quality directly affects model training, such that feature engineering can be regarded as setting the upper bound of achievable accuracy, whereas data quality sets its lower bound.

Raw TBM construction data contain a large amount of noisy data caused by operator behavior, equipment faults and other factors. Standardized data cleansing is therefore the basis for subsequent analysis^15,16^ and is essential for studying rock–machine interaction and extracting reliable characteristic parameters. There are currently two main strategies for computing and extracting features: (1) data-driven approaches, in which intelligent algorithms automatically select features based on statistical patterns; and (2) knowledge-driven approaches, in which features are selected based on physical and mechanical understanding and professional expertise^17^. Because TBM rock breakage is similar to in-situ indentation–shear testing, knowledge-driven approaches based on geotechnical expertise and TBM operating principles are generally considered more interpretable and are thus more widely used than purely data-driven methods^18–24^.In addition, data-driven approaches often suffer from limited interpretability, and variations in data collection types and volumes across projects make cross-project generalization challenging.

Most studies agree that cutterhead thrust *F*, cutterhead torque *T*, advance rate *v* and rotation speed *n* are the core parameters that reflect TBM mechanical status and rock-breaking performance^11,19,25–29^. To further reduce dimensionality, many researchers^17,30^ have linearly combined and transformed these basic parameters to derive rock mass boreability indexes, such as the field penetration index (FPI) and torque penetration index (TPI), which are then used as model inputs. Extensive research on FPI and TPI has demonstrated their feasibility for predicting rock mass quality^17,18,30–32^.

In practice, to make effective use of such characteristic parameters, they must be computed and processed in real time. In most current studies, FPI and TPI are directly computed from their definitions, which results in multiple values per boring cycle and considerable fluctuation, making it difficult to link them directly to rock mass quality. To address this, Chen et al.^17^ proposed a regression-based method for computing characteristic parameters, using the fitting coefficients to represent FPI and TPI for each cycle. This method is suitable for TBM tunnelling analysis under big-data conditions. However, its applicability is limited by the quality of curve fitting. Under certain geological conditions, such as fractured soft rock zones or ultra-hard rock segments, the relationships among parameters may become unclear and regularity is weak, leading to low-quality characteristic parameters that poorly reflect rock mass conditions.

Therefore, a key challenge is how to adapt to varying geological conditions, scientifically extract effective rock-breaking data, and achieve fast and accurate computation of FPI and TPI across different projects to support real-time evaluation of rock mass quality ahead of the TBM face.

Against this background, this study aims at rock mass quality identification during TBM advance. By coupling TBM disc cutter energy conversion with rock fracture characteristics, we identify high-efficiency rock-breaking segments, compute characteristic parameters across multiple projects and perform real-time rock mass quality prediction. The method is validated on TBM projects with different diameters and geological conditions, demonstrating its applicability and supporting the integration of big data and AI algorithms in TBM engineering for safer and more efficient construction. The main innovations of this study can be summarized as follows.

1. Limitations of existing FPI/TPI calculation methods in engineering applications.

Existing FPI/TPI-based methods are effective for rock mass quality prediction, but their applicability in modern TBM data environments remains limited because they often rely on direct calculation or project-specific regression, leading to unstable characteristic parameters, sensitivity to geological variability and data quality, and poor cross-project robustness.

(2) New capabilities of the proposed method for different engineering projects.

Unlike conventional methods that rely on large prior datasets and fixed penetration thresholds, this study identifies high-efficiency rock-breaking stages using local maxima of the FPI–t curve during tunnelling, enabling real-time characteristic-parameter computation with good timeliness. Validation across TBM projects with different diameters and geological conditions further demonstrates the generality of the proposed method.

## Engineering background and database

### Project background

**Fig. 1:**
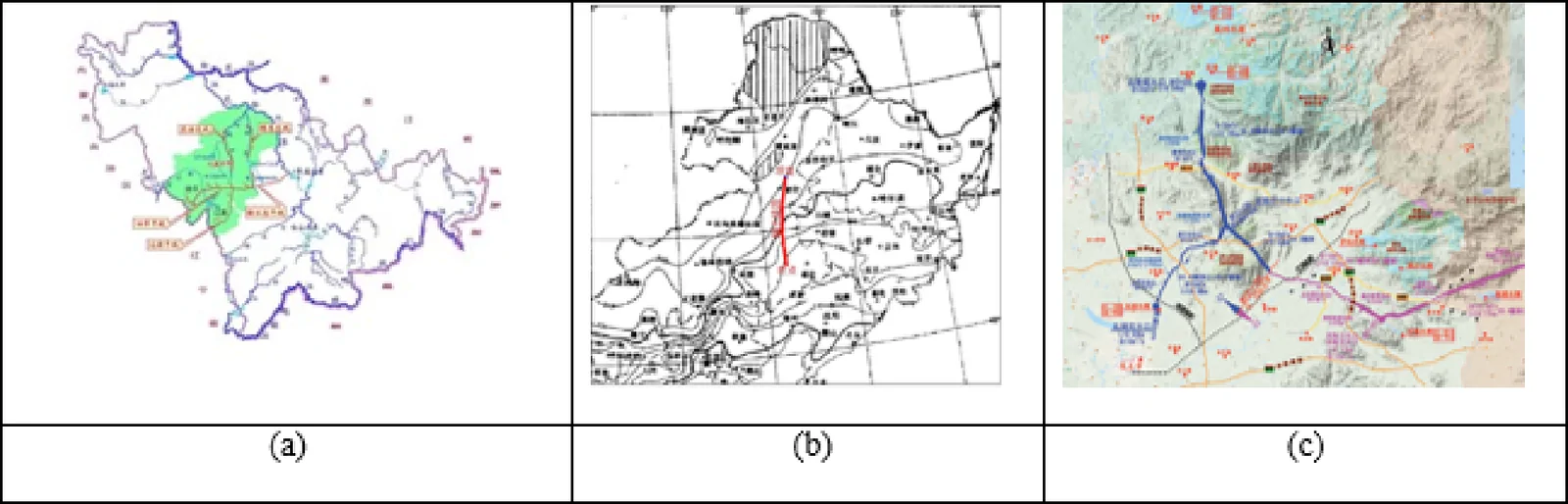
Schematic diagrams of TBM tunnelling in the three projects. (**a**)YS project, (**b**) YC project, (**c**) HB project. The maps were compiled by the authors based on non-confidential engineering geological information from the corresponding projects. Geological information related to the YS project has been previously reported in Ref.^24^.

**Fig. 2:**
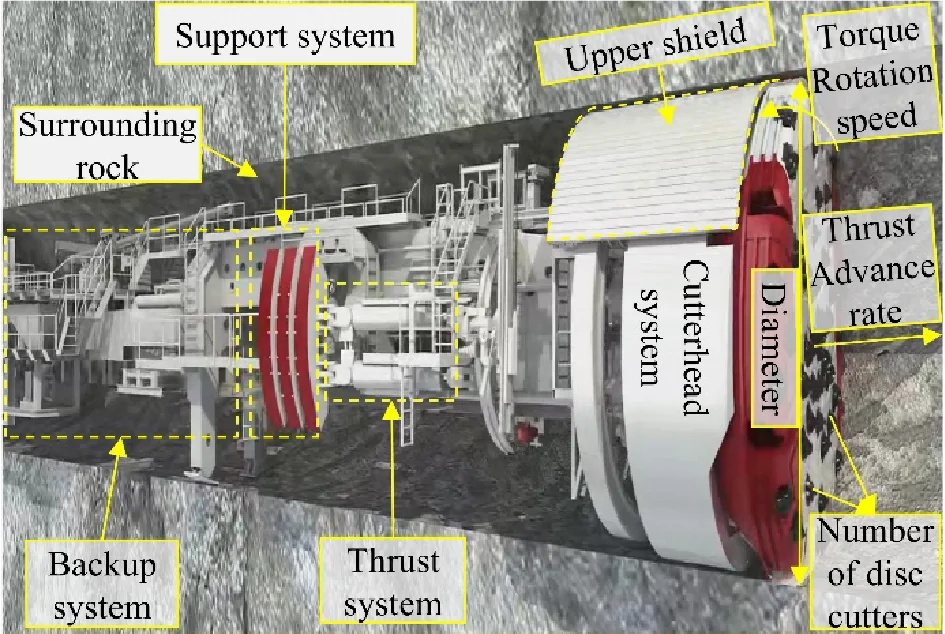
Schematic diagram of TBM design.

This study uses data from three TBM projects with different diameters and overburden depths: the Yinsong Water Diversion Project (YS), the Yinchao Water Diversion Project (YC), and the Huanbei Water Diversion Project (HB). Plan views are shown in Fig. 1, and the main TBM design and technical parameters are given in Fig. 2; Table 1.

**Table 1:** Main technical parameters of the TBMs used in the three projects.

Parameter	YS project	YC project	HB project
Cutterhead diameter/m	7.93	5.2	6.53
Maximum overburden/m	260	250	600
Number of disc cutters	56	34	41
Number of data channels	199	400	391
Maximum stroke/m	1.8	1.8	1.9
Rated torque/(kN m)	8410	3340	4322
Rated thrust/kN	23,260	11,340	21,991
Disentangling torque/(kN m)	12,615	4900	6483
Maximum advance rate/(mm min^−1^)	120	120	120
Maximum rotation speed/(r min^−1^)	7.6	11.45	8.4
Number of boring cycles	13,884	6419	253

The YS project employs an open-type TBM with a diameter of 7.93 m to construct a 17.45 km tunnel through surrounding rock classes II–V. Tunnelling started in May 2015 and was completed in January 2018. The YC project diverts water from the Chuoer River to the Xiliao River through a circular non-pressurized tunnel with a diameter of 5.2 m and a TBM-driven length of 18.8 km; tunnelling started in September 2020 and ended in December 2023. The HB project uses a TBM with a diameter of 6.53 m to excavate a 10.09 km tunnel, mainly through class III surrounding rock, followed by classes II, IV, and V. Tunnelling started on 17 October 2024 and was still ongoing at the time of this study.

### Database construction

#### Effective rock-breaking data

**Fig. 3:**
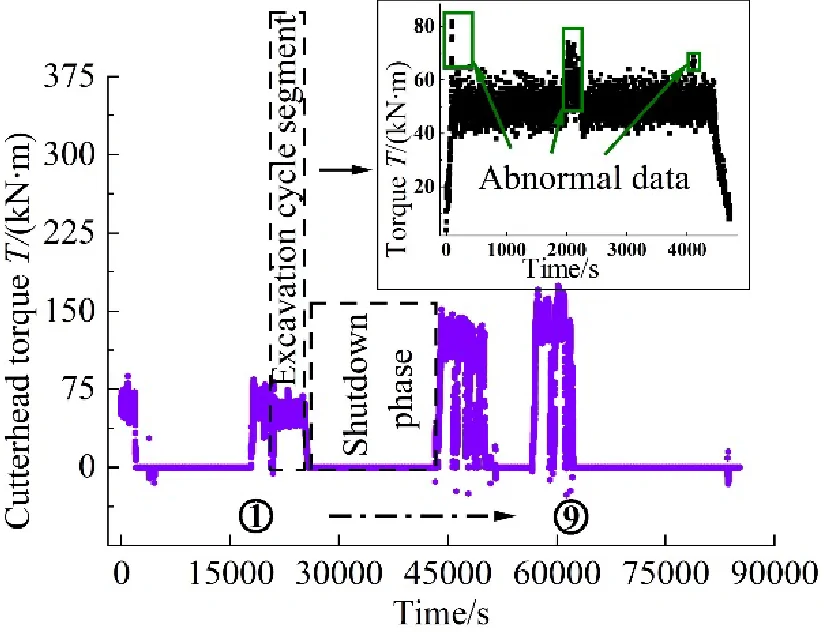
Tunnelling record of the YC project on 18 September 2020.

During TBM operation, hundreds of channels are recorded every second. Because of equipment faults, adverse geology, and other factors, downtime occupies a large proportion of the data^33^, and even working cycles contain many invalid records^16^. For example, on 18 September 2020, the YC project recorded 85,197 data rows but only nine valid boring cycles, with an effective working time of 17,196 s (20.2% of the total), as shown in Fig. 3. Rigorous preprocessing is therefore necessary.

The first preprocessing step is to identify the TBM operating state from cutterhead thrust, torque, advance rate, and rotation speed^22,29,34–39^, and divide the data into downtime and tunnelling stages. Abnormal conditions and invalid data are then removed using the rules summarized in Table 2. These methods mainly rely on parameter reasonableness and trends, but do not explicitly reflect the actual rock-breaking state. Further improvement in data quality is therefore still possible.

**Table 2:** Identification and handling of invalid TBM data.

Abnormal operation condition	Characteristics	Identification method	Processing method
Excessively short boring distance	Short cycle length	Length < 0.3 m	Discard the boring cycle
Short stoppage	Temporary standstill	Main tunnelling parameters drop to zero within a short period	Delete the corresponding data rows
Advance rate out of range	Abnormally high advance rate	Advance rate *v* > 120 mm·min^−1^	Set *v* = 120 mm min^−1^; delete out-of-range rows; remove outliers using the 3σ criterion
Data transmission anomaly^15^	Communication interruption or failure	Main tunnelling parameters remain unchanged for 10 s	Delete the corresponding data rows

**Fig. 4:**
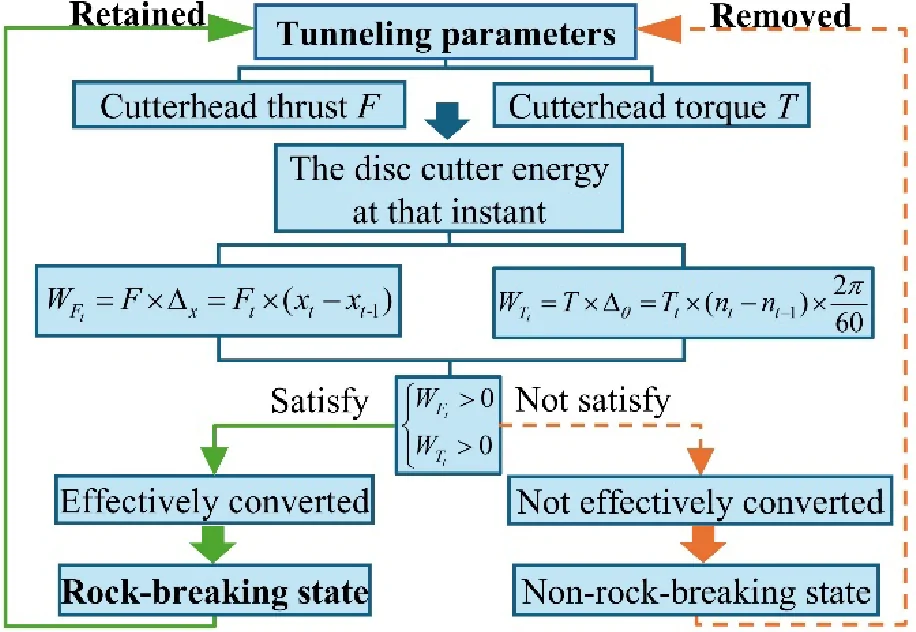
Flowchart of rock-breaking data filtering.

TBM rock breaking is analogous to a large-scale in situ torsional shear test^17^. Rock fragmentation is mainly produced by indentation from thrust and cutting by torque^40,41^. Focusing on a single disc cutter, this study evaluates whether cutter work is effectively converted into rock-breaking energy, as illustrated in Fig. 4. If either thrust work or torque work at a given time step is non-positive, the cutter cannot provide effective rock-breaking energy; the corresponding records are therefore treated as non-rock-breaking and removed. This criterion is necessary but not sufficient: $\:\boldsymbol{W}>0\:$does not necessarily imply efficient rock breaking. Low-efficiency cutting is retained and labeled as valid but low-efficiency data for subsequent modelling.

In Fig. 4, $\:{W}_{F}\:$and $\:{W}_{T\:}$denote the work done per second by the single-cutter thrust $\:{F}_{n}=F/N$and torque $\:{T}_{s}=T/N$, respectively (J), where * F*is cutterhead thrust (kN), * T*is cutterhead torque (kN m), and * N*is the number of cutters. Here, * t*is time (s), $\:{\Delta\:}x={x}_{t}-{x}_{t-1}$is the advance increment (mm), $\:{\Delta\:}\theta\:\:$is the rotation angle increment (rad), and $\:{n}_{t}$and $\:{n}_{t-1}$are the rotation speeds ($\:{\mathrm{r}\cdot\mathrm{m}\mathrm{i}\mathrm{n}}^{-1}$).

**Fig. 5:**
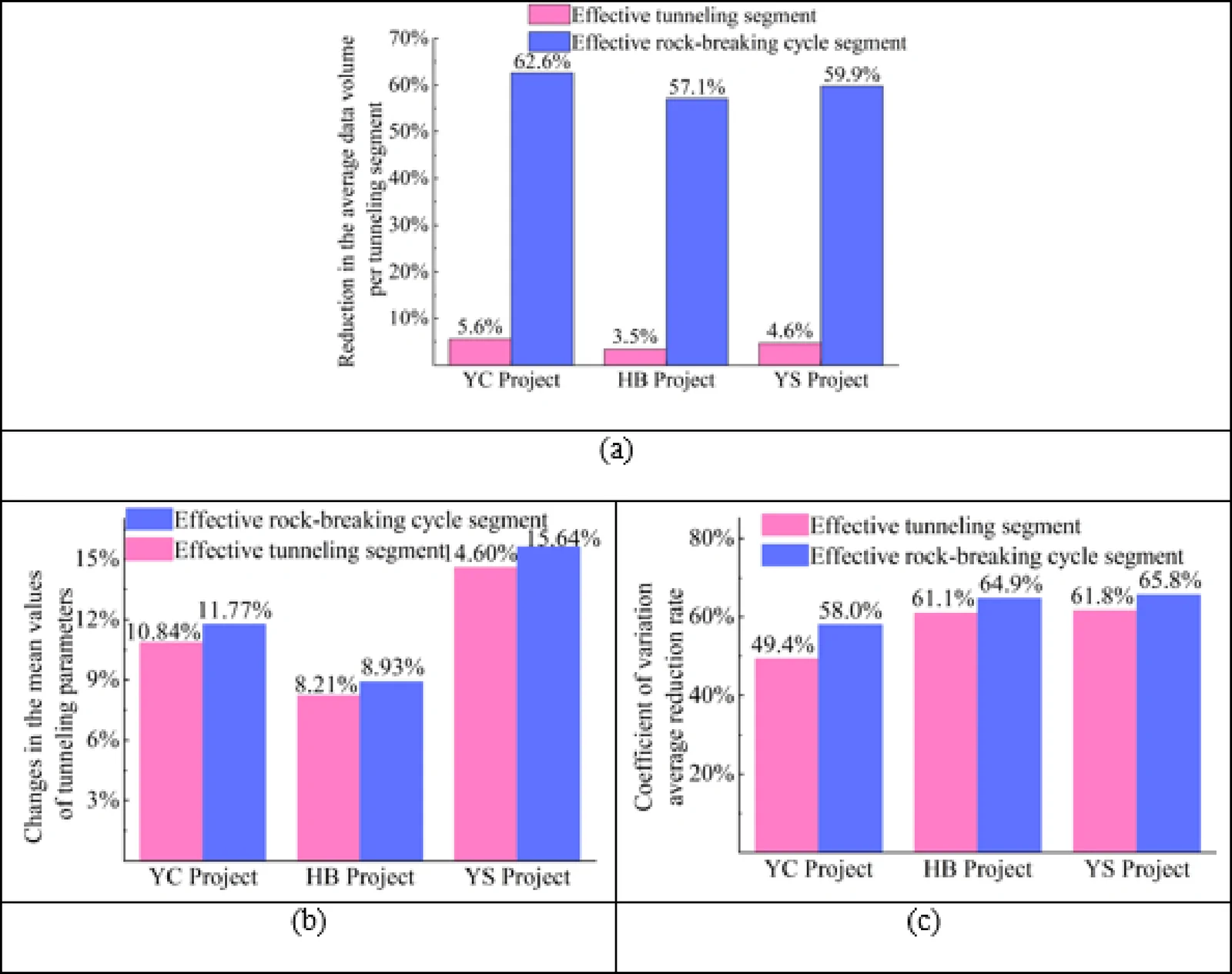
Comparison of parameter statistics before and after rock-breaking data filtering. (**a**) Reduction in data volume, (**b**) Changes in mean parameter values, (**c**) Reduction in coefficients of variation.

To verify applicability, conventional cleaning was first performed on YC, YS, and HB, followed by the proposed disc-cutter-energy filtering. This yielded 6005, 12,150, and 228 effective rock-breaking cycles for YC, YS, and HB, respectively. The changes in data volume and parameter statistics are summarized in Fig. 5.

As shown in Fig. 5, the proposed filtering method performs well in all three projects. After non-rock-breaking data are removed, the average number of data points per cycle decreases markedly, whereas the parameter means and coefficients of variation change only slightly. Thus, essential information is preserved while redundant or invalid records are reduced.

To further assess the robustness of using zero as the work threshold, the YC project was taken as an example. Thresholds of 0, 0.1, and 0.2 were applied to the effective rock-breaking cycle samples, and the numbers of samples in each surrounding rock class were compared, as shown in Table 3. Only slight changes were observed, indicating that small perturbations around zero do not materially affect class distributions; this further confirms that zero is both physically justified and robust, and it is therefore retained as the final work threshold.

**Table 3:** Statistics of sample numbers for each surrounding rock category in effective rock-breaking cycle segments under different thresholds (YC project).

Threshold	Samples			
	Class-II	Class-III	Class-IV	Class-V
0.0	1068	3387	945	605
0.1	1059	3382	945	602
0.2	1048	3382	945	585

#### High-efficiency rock-breaking stage

**Fig. 6:**
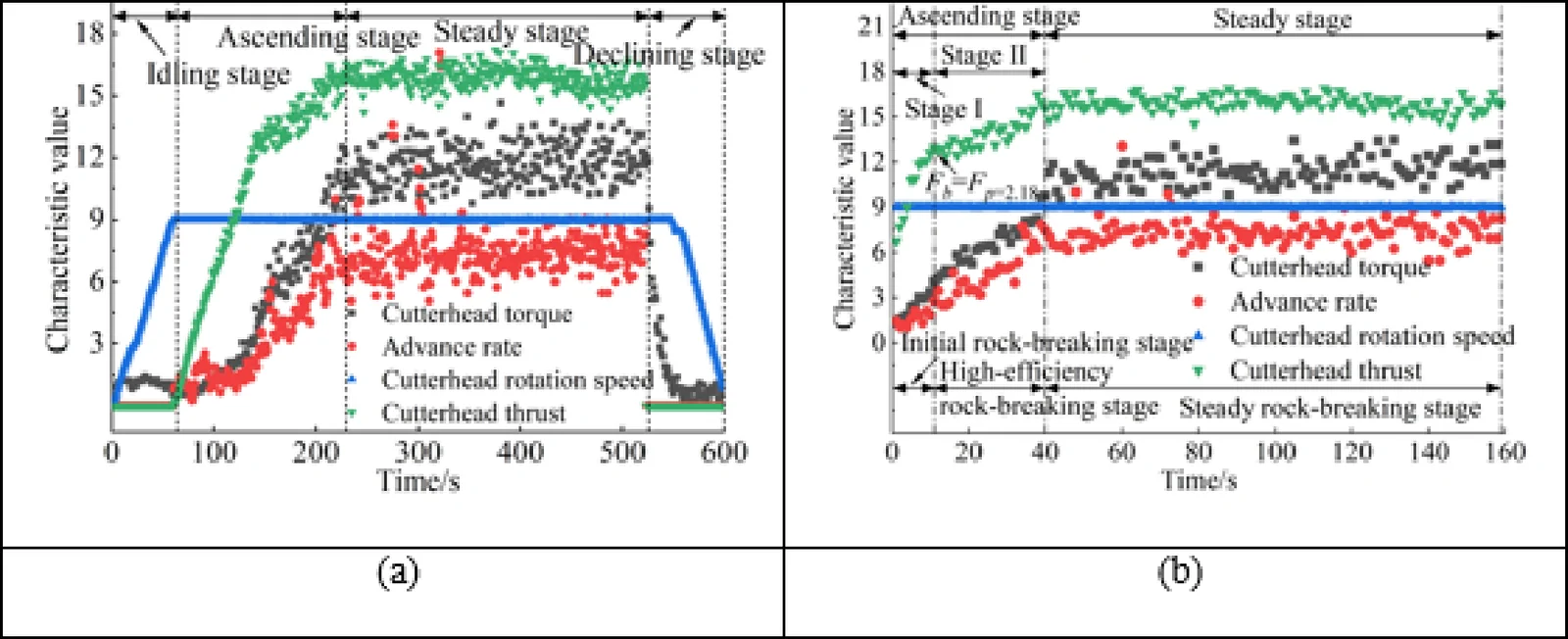
Comparison of tunnelling data before and after rock-breaking data filtering. (**a**) Original boring cycle and tunnelling stages, (**b**) Effective rock-breaking cycle and rock-breaking stages.

Within a complete boring cycle, the TBM typically passes through idle advance, initial contact with the face, stable high-efficiency rock breaking, and shutdown^18^. Based on the evolution of tunnelling parameters, a cycle can be divided into idling, ascending, stable, and descending stages^16^. Taking the YC project at chainage 59,957.4 m as an example, the segmentation based on raw data is shown in Fig. 6a.

Some studies further subdivide the ascending stage. Wang et al.^34^ divided it into two parts: an initial stage of rapid thrust and torque growth, corresponding to initial tool-rock contact and rock breakage, and a second stage beginning when the applied load exceeds the critical rock-breaking force $\:{\boldsymbol{F}}_{\boldsymbol{b}}$, after which high-efficiency rock breaking starts. In practice, because direct in situ determination of rock parameters is difficult, $\:{\boldsymbol{F}}_{\boldsymbol{b}}$is usually approximated by a penetration-per-revolution threshold^32,34^.

In this study, non-rock-breaking data are first removed using the cutter-energy criterion in “Effective rock-breaking data”, and the high-efficiency rock-breaking stage is then identified. The end of the ascending stage is determined using histogram statistics of tunnelling parameters^40^(We construct an equal-width histogram (B bins) of the smoothed tunnelling parameter (W-point centered moving average). The modal bin is identified, and its upper edge is used as a threshold. The stable-start is the first sample whose raw value exceeds $\:\mathrm{T}\mathrm{-}\mathrm{m}$, subject to jitter and trend gates based on relative change-rate thresholds $\:\delta_{\mathrm{j}}$and $\:\delta_{\mathrm{t}}$. For multiple parameters, candidate start indices are fused by IQR-based outlier removal and the median index). Figure 6b shows the resulting segmentation for the same cycle after rock-breaking filtering.

## Computation of rock mass characteristic parameters based on rock-breaking features

### Rock mass boreability indexes

**Fig. 7:**
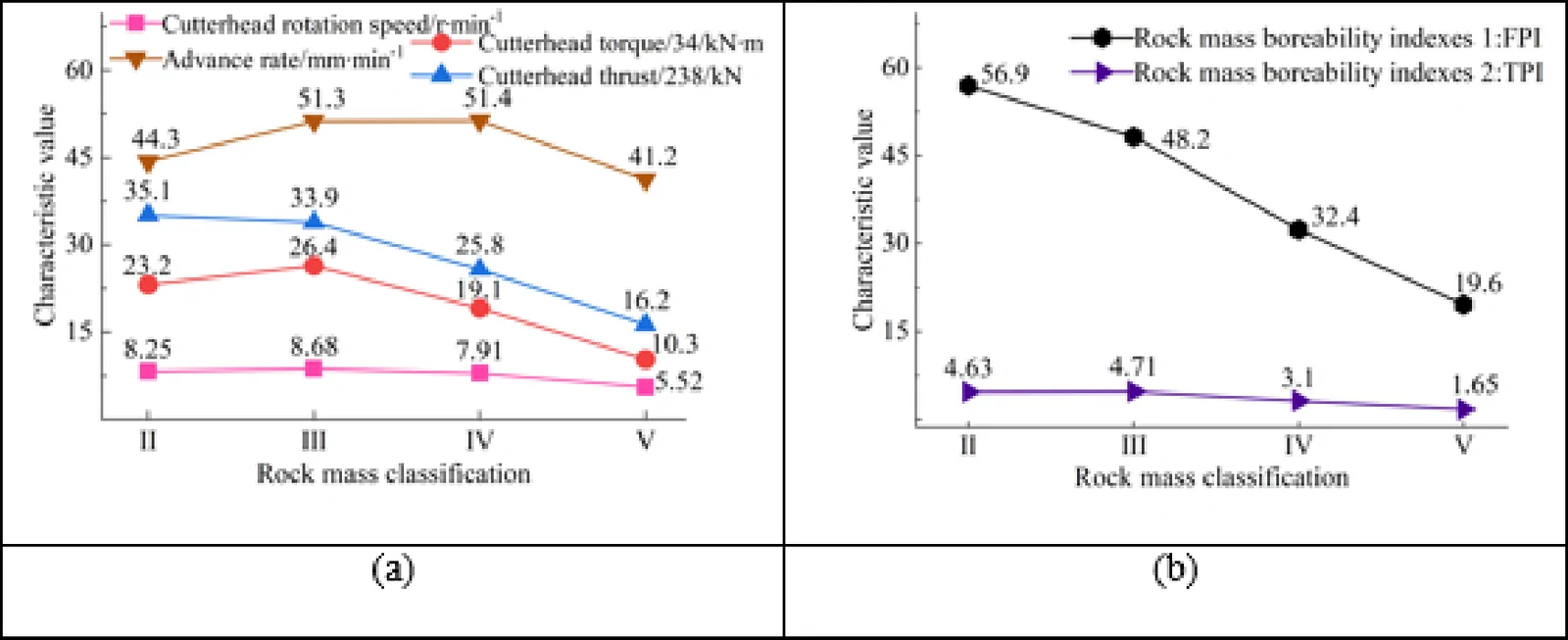
Distribution of tunnelling parameters and rock mass boreability indexes across rock mass classes in the YC project. (**a**) Tunnelling parameters, (**b**) Boreability indexes.

Rock mass quality is a key basis for identifying adverse geology and optimizing TBM performance^11,42^. In practice, thrust and torque are often used directly to infer rock conditions. However, analysis of the YC project shows that raw tunnelling parameters do not correlate clearly with rock mass classes overall (Fig. 7a).

To overcome this limitation, boreability indexes have been proposed. FPI and TPI characterize rock-mass behavior by integrating the rock-machine interaction process^17,30^ and are defined as:

$${\mathrm{FPI}}=\frac{{{F_n}}}{p}=\frac{{F/N}}{{Pr/RPM}}$$

$${\mathrm{TPI}}=\frac{{{T_s}}}{p}=\frac{{T/N}}{{Pr/RPM}}$$

where *Fn* is the average single-cutter thrust (kN); *F* is the total cutterhead thrust (kN); *N* is the number of cutters; *Pr* is the penetration rate (mm min^− 1^); RPM is the cutterhead rotation speed (r min^− 1^); and *T* and *Ts* are the total and single-cutter torques, respectively (kN m).

Using these definitions, the mean FPI and TPI of each effective rock-breaking cycle in the YC project were calculated, and their distributions across rock mass classes are shown in Fig. 7b. Compared with the raw tunnelling parameters, FPI and TPI exhibit clearer and generally positive correlations with rock mass quality. This is further supported by the inter-class difference analysis in Table 4, which shows that the mean inter-class variation increases from 19.35% for the raw parameters to 28.38% for FPI/TPI, indicating that the boreability indexes are more informative for distinguishing rock mass quality.

**Table 4:** Inter-class difference test.

Input parameters	Parameter	Variation ranges between rock mass classes			
		Variation (Class II → III)	Variation (Class III → IV)	Variation (Class IV → V)	Mean
Raw tunnelling parameters	Cutterhead torque	5.21%	8.87%	30.21%	14.77%
	Cutterhead thrust	13.79%	27.65%	46.07%	29.17%
	Advance rate	3.42%	23.89%	37.21%	21.51%
	Cutterhead rotational speed	15.80%	0.19%	19.84%	11.95%
	Average	9.56%	15.15%	33.34%	19.35%
Rock-breakability indices	FPI	15.29%	32.78%	39.51%	29.19%
	TPI	1.73%	34.18%	46.77%	27.56%
	Average	8.51%	33.48%	43.14%	28.38%

### Fitting-based parameter calculation method

Direct calculation of characteristic parameters (FPI/TPI) is straightforward but often results in strong fluctuations^32^. To address this issue, Chen Zuyu’s team proposed a regression-based method in which characteristic parameters are computed by fitting tunnelling data^34^. In this framework, the coefficients of the thrust–penetration (* F*–* p*) relationship, * a* and * b*, are used to characterize FPI: $\:a\:$denotes the thrust increment per unit penetration during the stable rock-breaking stage, reflecting the integrated boreability of the surrounding rock under sustained cutting, whereas * b*, with $\:F\approx\:b\:$as $\:p\to\:0$, represents the initial breakage threshold associated with tool–rock engagement and crack initiation. The fitted torque–penetration (* T*–* p*) relationship is used to characterize TPI. In this way, each boring cycle is uniquely represented by a single parameter set (*a*, *b*, TPI).

**Fig. 8:**
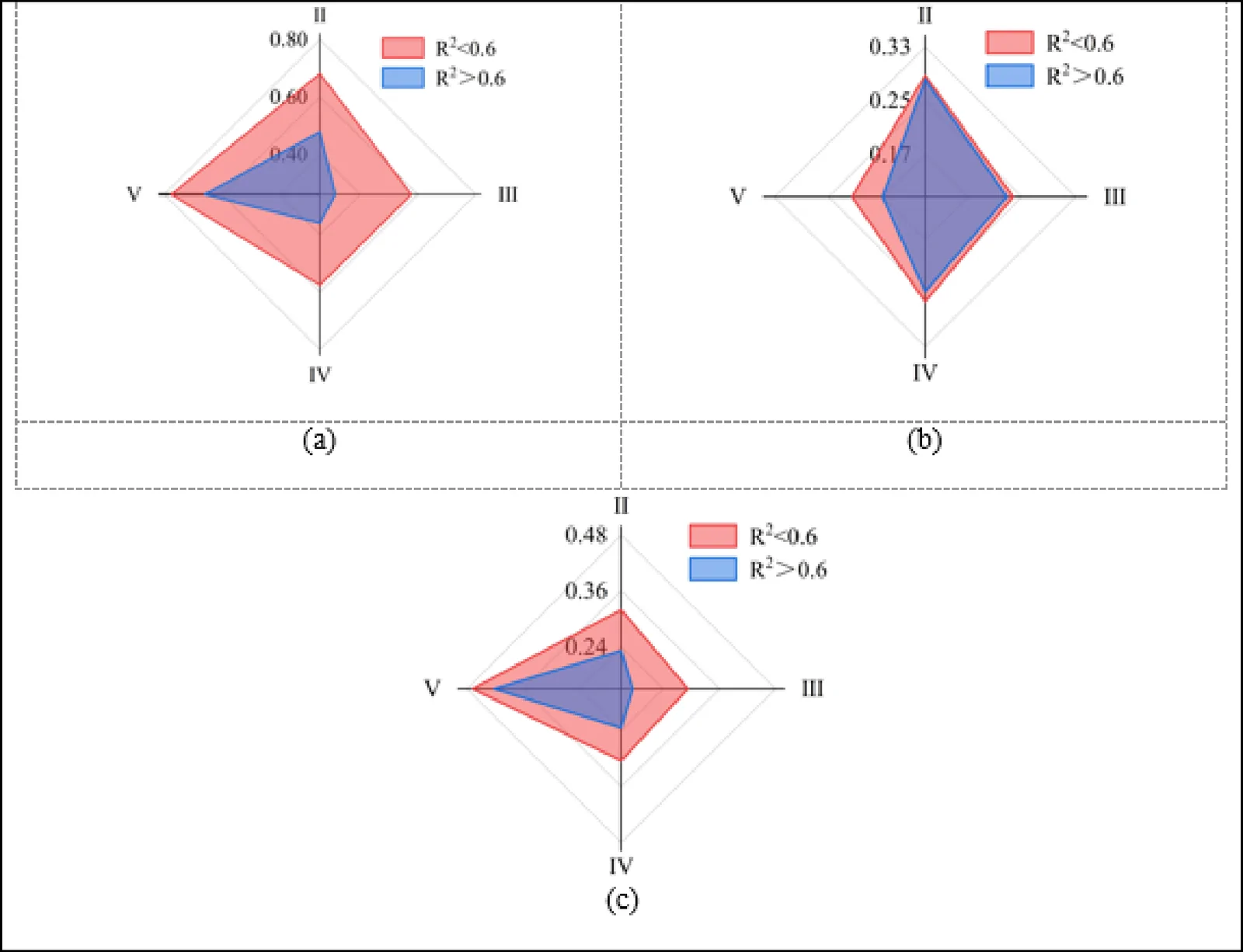
Coefficients of variation of *a*, *b* and TPI for different levels of goodness-of-fit. (**a**) Distribution of the CV of *a* across different surrounding rock categories. (**b**) Distribution of the CV of *b* across different surrounding rock categories. (**c**) Distribution of the CV of TPI across different surrounding rock categories.

The effectiveness of this method depends strongly on fitting quality. To illustrate this, 2299 effective rock-breaking cycles from the YC project (chainages 66,059–55,721 m and 25,916–25,525 m) were analyzed. The characteristic parameters were computed by curve fitting, and the coefficients of variation (CVs) of * a*, * b*, and TPI were compared between cycles with goodness-of-fit ($\:{R}^{2}$) lower than 0.6 and those with $\:{R}^{2}$higher than 0.6. The results are shown in Fig. 8. As $\:{R}^{2}\:$increases, the CVs of * a*, * b*, and TPI decrease across all rock mass classes. This indicates that higher fitting quality leads to lower within-class variability and, therefore, higher-quality characteristic parameters. In other words, the quality of curve fitting directly determines the quality of the derived characteristic parameters. Accurate selection of fitting data is therefore critical for effective regression-based parameter calculation.

As discussed in “High-efficiency rock-breaking stage”, a key issue is how to determine the boundary of the high-efficiency rock-breaking stage. Existing studies usually rely on project-specific statistics and adopt a fixed penetration threshold (e.g., *p* = 2 mm·rev^− 1^) to approximate the rock-breaking force $\:{F}_{b}$and identify the boundary of the ascending stage. However, because geological conditions vary across projects, this fixed penetration-based criterion is often unsuitable for cycles with strongly fluctuating penetration under special conditions.

**Fig. 9:**
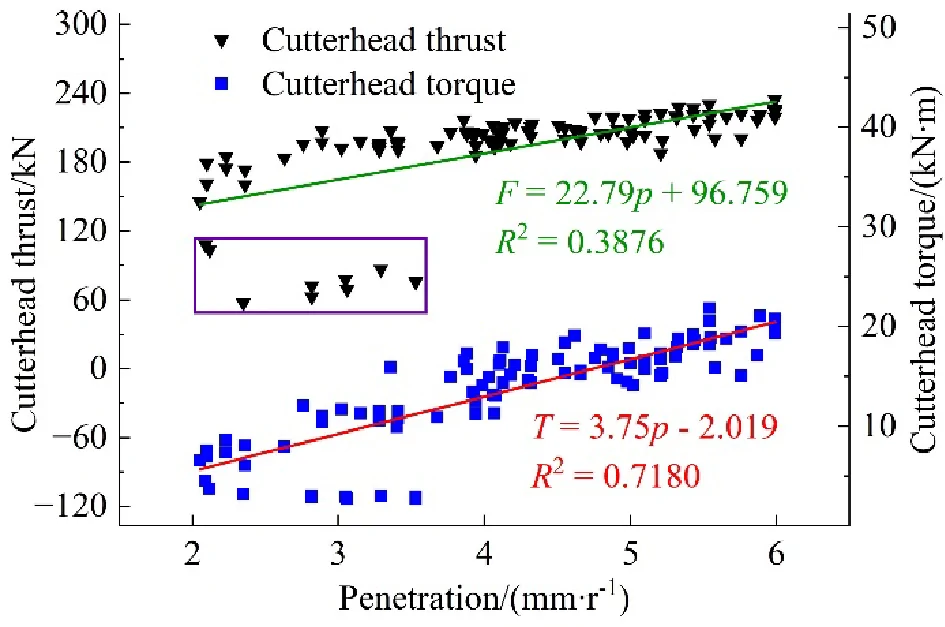
Correlation of parameters in the high-efficiency rock-breaking stage extracted using the penetration-threshold method.

For example, in the YC project at chainage 65,823.2 m, penetration ranges from 2 to 3.5 mm rev^− 1^ and contains obvious outliers. As shown in Fig. 9, the * F*–* p* and * T*–* p* relationships extracted by the penetration-threshold method exhibit poor goodness-of-fit. In addition, at the initial stage of construction, the accumulated data may be insufficient to determine a reliable project-specific threshold.

### Knowledge-driven feature parameter extraction method

**Fig. 10:**
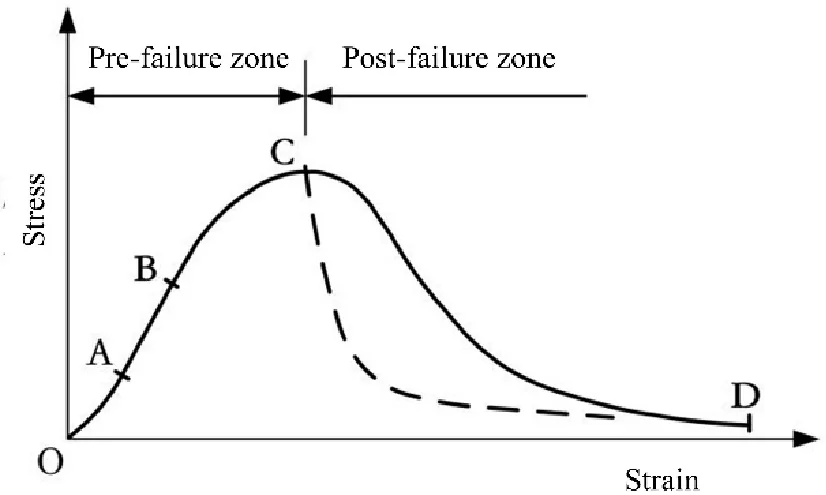
Stress–strain curve of rock failure process^48^.

The stress–strain curve of rock failure (Fig. 10) shows that stress first compacts the rock and increases its load-bearing capacity; once the ultimate strength is exceeded, the strength and elastic modulus decrease and macro-failure occurs. Similar progressive failure characteristics have also been reported in studies on deep hard rock fracture and rock mass kinematic evolution^43,44^. Recent studies further suggest that this macroscopic response is controlled by heterogeneous microstructure evolution, fracture architecture, and stress-dependent nonlinear deformation behavior^45–47^.

During TBM cutting with disc cutters, the rock–cutter interaction follows a similar progressive process. At the initial penetration stage, pores and microcracks are compacted, so the cutter thrust generally rises with penetration. As penetration continues, damage accumulates and cracks propagate beneath the cutter, and the thrust reaches its peak. Once unstable macro-fracture and rock chipping occur, the local load-bearing capacity decreases, leading to a reduction in thrust. Therefore, the thrust evolution described by the model reflects the progressive damage evolution of the rock mass, rather than merely a phenomenological variation in cutter load.

**Fig. 11:**
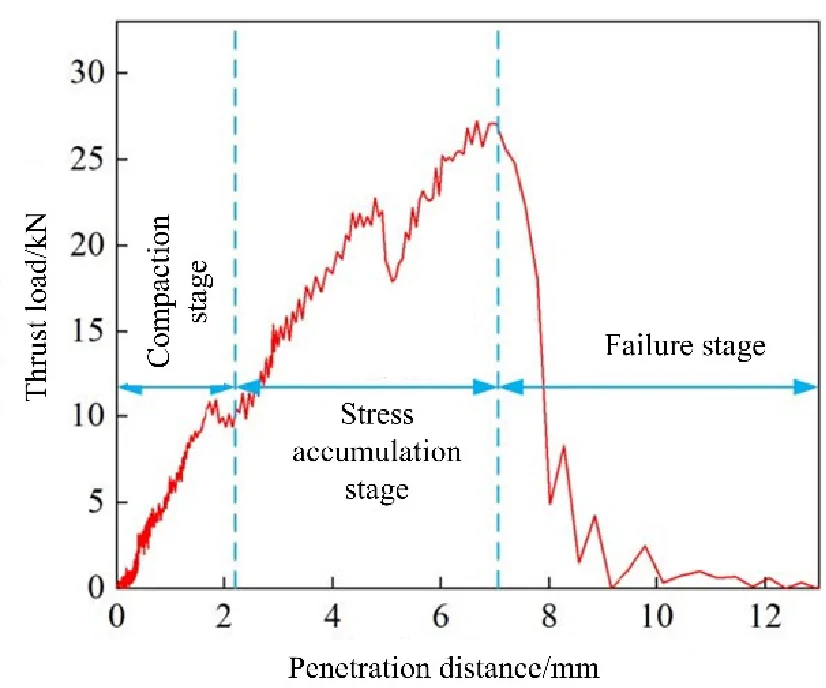
Thrust load–penetration distance curve obtained from disc cutter rock-breaking test^48^.

This trend has been confirmed by the disc-cutter indentation tests conducted by Wang^48^ (Fig. 11) and by the TBM rock-breaking model tests reported by Jiang et al.^49^. Accordingly, thrust evolution can be used to distinguish different rock-breaking stages: in the ascending phase, FPI increases with penetration, whereas its decrease indicates the onset of unstable failure. The turning point of the FPI–time (FPI–t) curve therefore marks the transition from stable damage development to unstable macro-fracture propagation, indicating that FPI evolution provides a model-consistent mechanical basis for identifying the onset of the high-efficiency rock-breaking stage, rather than serving as a purely empirical criterion.

On the basis of the above analysis of thrust–rock fragmentation characteristics, the local maximum of the FPI–*t* curve is adopted as the onset of the high-efficiency rock-breaking stage in the ascending phase. The implementation includes signal smoothing and local peak detection. The signal is first segmented using a 15-point window (window_size = 15). A candidate onset is identified when two consecutive segments satisfy the following pattern: the mean of the first 7 points is greater than the global mean, whereas the mean of the last 7 points is lower than the global mean. Then, over the full sequence, a 3-point moving average is iteratively updated with a step size of 3, and the index corresponding to the maximum 3-point mean is selected as the reference point.

To evaluate robustness, Monte Carlo simulations were conducted under different sampling rates (1/2/5/10 Hz) and noise levels (Gaussian noise plus random spikes). The results show that the proposed detection strategy remains stable over the tested range: Under a ± 5 s tolerance criterion, the miss rate is zero for all tested combinations, and the success rate remains nearly 100%, decreasing slightly to 0.995 only when fs = 1 Hz and σ = 1.0. Although the localization error increases monotonically with noise level, it remains within approximately 0.3–1.5 s. These results confirm that the local-maximum method is robust to practical variations in sampling frequency and noise level.

**Fig. 12:**
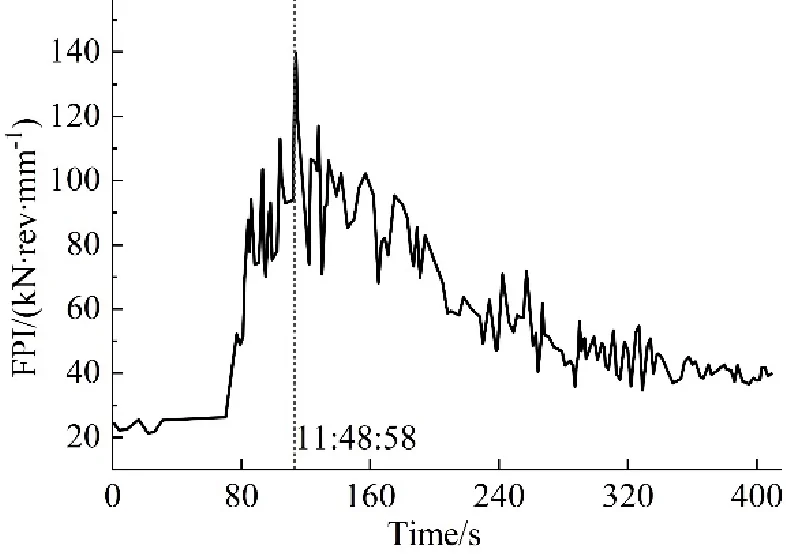
Schematic illustration of selecting the high-efficiency rock-breaking stage based on the FPI–t curve.

**Fig. 13:**
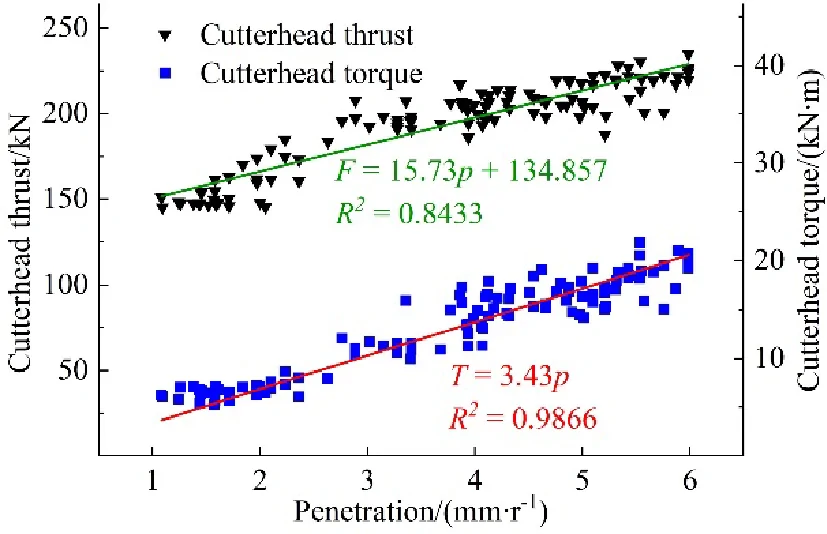
Relationships between cutterhead torque and penetration and between cutterhead thrust and penetration for the selected high-efficiency rock-breaking data.

Taking the YC project at chainage 65,823.2 m as an example, Fig. 12 shows the FPI–* t* curve and the selected data segment to the right of the local maximum. The corresponding * T*–* p* and * F*–* p* relationships are shown in Fig. 13. Compared with Fig. 9, the anomalous penetration data are effectively removed, and the goodness-of-fit of the * F*–* p* and * T*–* p* regressions improves from 0.3876 to 0.7180 to 0.8433 and 0.9866, respectively.

Among the 6005 boring cycles in the YC project, 1400 cycles (23.31%) exhibit strong penetration fluctuations. For these cycles, the high-efficiency rock-breaking stage was extracted using both the penetration-threshold method and the proposed FPI–$\:\boldsymbol{t}$ local-maximum method. The resulting characteristic parameters were then statistically compared, and the results are summarized in Table 5.

**Table 5:** Comparison of different methods for extracting the high-efficiency rock-breaking stage in problematic cycles.

Criterion for extracting high-efficiency rock-breaking stage	CV			Goodness-of-fit	
	a	b	TPI	FPI	TPI
Penetration > 2 mm·rev⁻¹	0.568	0.353	0.388	0.355	0.602
Local maximum of FPI–t curve	0.531	0.245	0.335	0.605	0.753
Change (%)	− 6.39%	− 30.51%	− 13.73%	+ 70.47%	+ 25.17%
Average change	− 16.88%			+ 47.82%	

Table 5 indicates that, compared with the penetration-threshold method, the local-maximum method based on the FPI–t curve reduced the average coefficient of variation of the characteristic parameters by 16.88% and improved the average goodness of fit by 47.82%. This suggests that, in abnormal cycles with significant penetration fluctuations, the proposed method can extract more stable characteristic parameters with better fitting performance. From an engineering perspective, these improvements mean that the extracted parameters more accurately reflect the actual rock-breaking state, especially in sections with poor surrounding rock conditions or large parameter fluctuations. As a result, the method can reduce fitting errors and feature distortion, lower the risk of misclassification in real-time surrounding rock classification, and support earlier and more accurate identification of poor surrounding rock zones for tunneling parameter adjustment and safety decision-making.

To further assess the consistency of this improvement, the per-sample improvement distribution for the 1400 cycles was summarized and a paired Wilcoxon signed-rank test was performed (Table 6). The median absolute improvement is 0.2409 (IQR: 0.2068), and the median relative improvement is 62.05% (IQR 120.41%). The Wilcoxon test gives $\:p=8.67\times\:{10}^{-209}$, confirming that the improvement in goodness-of-fit is systematic rather than a random fluctuation.

**Table 6:** Distributional statistics of per-sample improvements and paired Wilcoxon test.

Items	Metric definition	Median	IQR	Sample size(*n*)
Distribution (absolute improvement)	diff = new − old	0.2409	0.2068	1400
Distribution (relative improvement)	(new − old)/|old| × 100%	62.05%	120.41%	1400
Significance test	Paired Wilcoxon signed-rank	*p* = 8.67 × 10⁻^209^	–	1400

In summary, the proposed knowledge-driven real-time feature-parameter extraction method, derived from the analysis of disc-cutter thrust–rock fragmentation characteristics, consists of three steps: removing non-rock-breaking data based on effective cutter-work energy conversion, identifying the high-efficiency rock-breaking stage from the local maximum of the FPI–*t* curve, and fitting the *F*–*p* and *T*–*p* relationships to obtain the characteristic parameters * a*, * b*, and TPI.

## AI-based rock mass quality prediction with cross-project transferability

### Model development and validation for the YC project

#### Comparative evaluation of AI-driven machine learning models

**Fig. 14:**
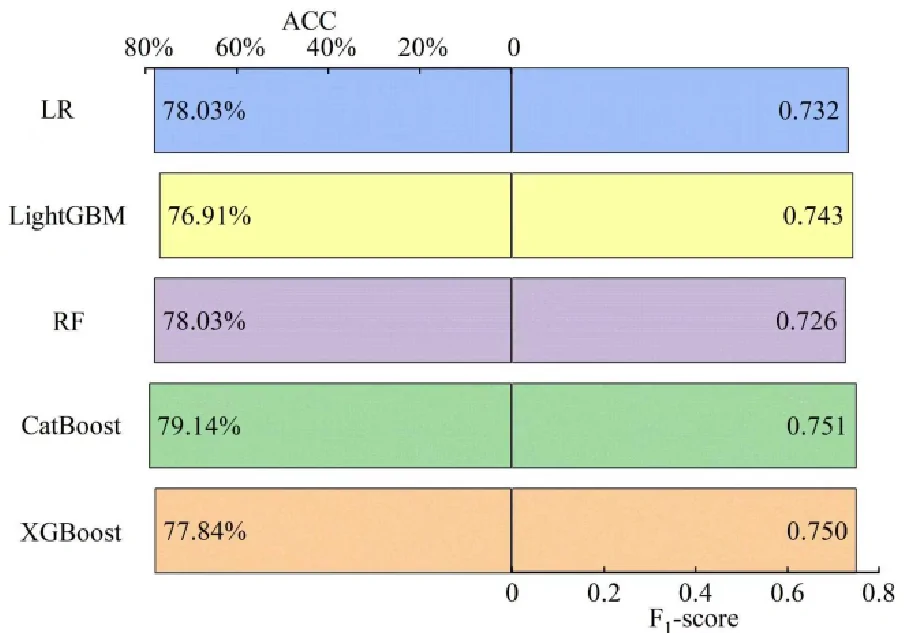
Comparison of rock mass quality prediction performance for different ML models.

In this subsection, multiple AI-driven machine learning models are developed and systematically compared to evaluate predictive performance and identify the most suitable model configuration. To evaluate the proposed knowledge-driven characteristic-parameter computation method, the 6,005 effective rock-breaking cycles of the YC project were used to calculate the input features $\:\boldsymbol{a}$, $\:\boldsymbol{b}$, FPI goodness-of-fit, TPI, and TPI goodness-of-fit. Five machine-learning algorithms were considered for rock mass quality prediction: XGBoost, CatBoost, Random Forest (RF), LightGBM, and Logistic Regression (LR). Accuracy (ACC) and F1-score were used as evaluation metrics, and the results are shown in Fig. 14. CatBoost achieves the best overall accuracy and the clearest advantage in F1-score. It was therefore selected as the base model for this study.

**Fig. 15:**
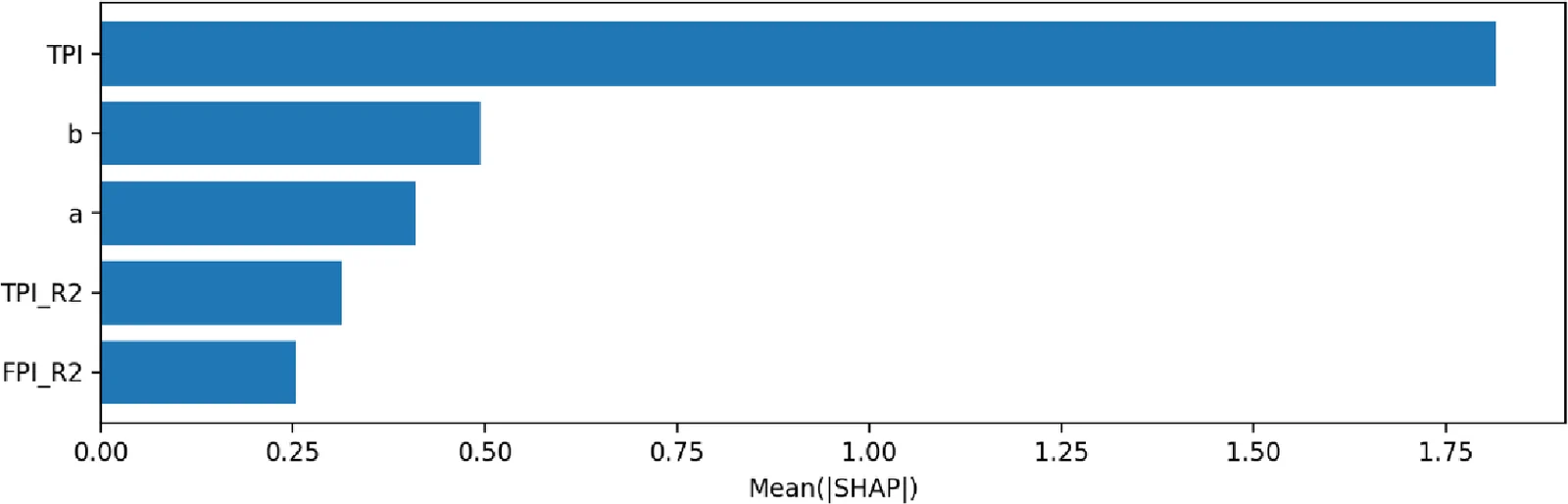
Global feature importance of the CatBoost model based on Mean(|SHAP|).

Furthermore, to substantiate the knowledge-driven nature of the proposed framework and improve interpretability, SHAP (Shapley Additive Explanations) was used to quantify the contributions of the input features to the CatBoost predictions. As shown in Fig. 15, TPI has the highest global importance (Mean(|SHAP|) = 1.8154), followed by * b*(0.4939), * a*(0.4099), TPI_R2 (0.3139), and FPI_R2 (0.2540). This indicates that the model mainly relies on the knowledge-based information encoded by TPI, while * a*and * b*provide complementary contributions. In contrast, the relatively low contribution of the R^2^-type goodness-of-fit indicators reduces the risk that the model relies on fitting quality or other potential spurious correlates.

#### Model construction, optimization and testing

CatBoost is a gradient-boosting algorithm that uses ordered target statistics and ordered boosting to reduce target leakage and gradient bias. Its symmetric tree structure improves stability and generalization in practice^50–52^.The CatBoost model contains multiple hyperparameters. Among them, iterations and learning_rate control the learning rate and convergence process; depth and l2_leaf_reg control model complexity and prevent overfitting; and random_strength and bagging_temperature adjust model randomness to enhance generalization.

To maximize the predictive power of CatBoost, the input feature vectors are first filtered based on the conclusions of “Fitting-based parameter calculation method”: the goodness-of-fit of the FPI regression is used as a criterion for screening characteristic parameters. The parameters computed in “Comparative evaluation of AI-driven machine learning models” are grouped according to FPI goodness-of-fit, and four prediction models are built for different ranges of goodness-of-fit.

**Fig. 16:**
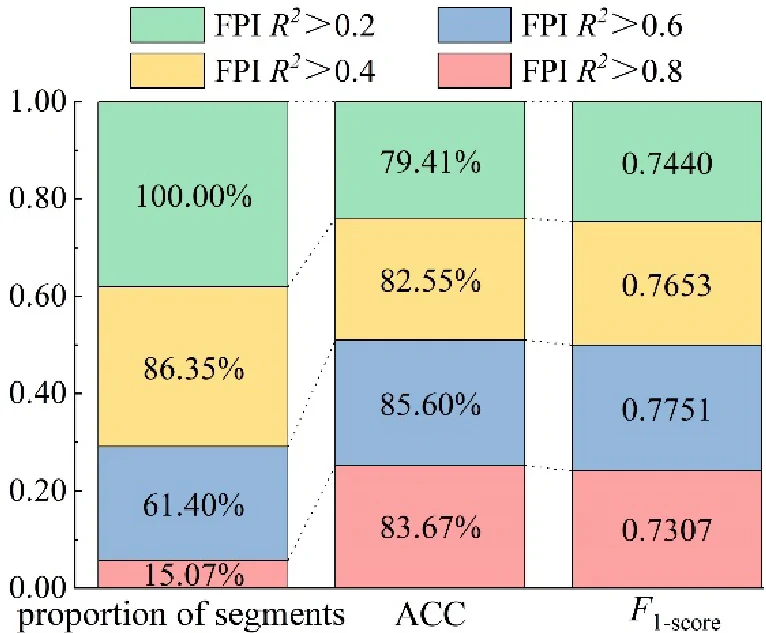
Rock mass quality prediction performance for cycle groups with different FPI goodness-of-fit levels.

Each model was evaluated using stratified five-fold cross-validation. Hyperparameters were optimized by Bayesian optimization with Optuna TPE (TPESampler(seed = 42)) to maximize the five-fold macro-F1. The search space was: iterations [300, 2000], learning_rate [0.01, 0.3] (log), depth [4, 10], l2_leaf_reg [1, 20] (log), random_strength [0, 20], and bagging_temperature [0, 1], with bootstrap_type = Bayesian. Thirty trials were run for each dataset with fixed seeds (42), single-thread execution (thread_count = 1), and early stopping (od_wait = 100, use_best_model=True). The average metric across the five-folds serves as the objective function to be maximized, and the hyperparameter combination that achieves the best validation performance is selected as the optimal setting. The corresponding sample numbers and prediction results are shown in Fig. 16.

As the goodness-of-fit increases, both accuracy and F1-score generally improve, while the number of available cycles decreases. The best performance is obtained when the FPI goodness-of-fit exceeds 0.6. When it exceeds 0.8, the number of cycles drops sharply and prediction accuracy declines because of insufficient training data. Therefore, 0.6 is selected as the boundary between high- and low-quality feature sets. The optimal hyperparameters of the YC CatBoost model are listed in Table 7, and the final model achieves an accuracy of 85.60%. Five-fold cross-validation further indicates stable performance, with an ACC of 0.8560 ± 0.0083 and an F1-score of 0.7751 ± 0.0147. Detailed results are summarized in Table 8.

**Table 7:** Optimal hyperparameter combination of the best CatBoost model for the YC project.

Hyperparameter	Description	Optimal value
Iterations	Number of boosting iterations	786
Learning_rate	Learning rate	0.2950
Depth	Maximum tree depth	7
l2_leaf_reg	L2 regularization coefficient	2.4733
Random_strength	Randomness of feature importance	3.7809
Bagging_temperature	Sampling temperature	0.2179

**Table 8:** Summary of five-fold cross-validation results for the YC, YS, and HB projects.

Project	Metric	Fold 1	Fold 2	Fold 3	Fold 4	Fold 5	Mean	SD	95% CI
YC	ACC	0.8667	0.8450	0.8602	0.8571	0.8511	0.8560	0.0083	0.8457–0.8664
	F1-score	0.7727	0.7675	0.7725	0.8004	0.7624	0.7751	0.0147	0.7568–0.7934
YS	ACC	0.8669	0.8692	0.8623	0.8634	0.8621	0.8648	0.0031	0.8609–0.8687
	F1-score	0.8464	0.8551	0.8394	0.8475	0.8422	0.8461	0.0060	0.8387–0.8535
HB	ACC	0.8407	0.8148	0.8431	0.8407	0.8889	0.8456	0.0268	0.8123–0.8789
	F1-score	0.8120	0.8767	0.8723	0.8381	0.8910	0.8580	0.0322	0.8180–0.8980

#### Model application for scheme validation and comparison

To further validate the proposed characteristic parameter computation method, three calculation schemes are compared using the YC project data and the optimized CatBoost model from Section.

Method 0: No data preprocessing; high-efficiency rock-breaking stage identified by the local maximum of the FPI–$\:\boldsymbol{t}$ curve; characteristic parameters computed by regression.

Method I: Conventional preprocessing; high-efficiency stage identified by the penetration-threshold method(*p* = 2 mm·rev^−1^); characteristic parameters computed by regression.

Method II: Conventional preprocessing; high-efficiency stage identified by the local maximum of the FPI–$\:\boldsymbol{t}$ curve; characteristic parameters computed by regression.

Method III (proposed): Rock-breaking data preprocessing to obtain effective rock-breaking cycles; high-efficiency stage identified by the local maximum of the FPI–$\:\boldsymbol{t}$ curve; characteristic parameters computed by regression.

After filtering using the FPI goodness-of-fit threshold of 0.6, 858, 833, 2020 and 1919 groups of characteristic parameters are obtained by Methods I, II and III, respectively. A total of 170 common cycles are randomly selected as the test set (stratified random sampling by rock mass class), and the remaining cycles are used for training. Overall accuracy (ACC) and *F*_*1*_-score are used as evaluation metrics. The results are summarized in Tables 9 and 10.

**Table 9:** Comparison of different characteristic parameter computation methods in the YC project.

Method	Method for calculating input feature parameters		Number of high-quality feature sets	Prediction of surrounding rock quality			
	Data preprocessing result	Criterion for starting high-efficiency rock-breaking stage		Training set/segments	Test set/segments	Accuracy ACC/%	*F*_*1-*_score
Method 0	Original cycles	Local maximum of FPI–t curve	858	688	170	79.17	0.8016
Method I	Valid boring cycles	Penetration > 2 mm·rev^−1^	833	663		80.59	0.7699
Method II		Local maximum of FPI–t curve	2020	1850		82.94	0.8134
Method III	Effective rock-breaking cycles	Local maximum of FPI–t curve	1919	1749		84.12	0.8244

**Table 10:** Detailed predictive performance metrics for the YC project.

Method	Rock mass class							
	II		III		Ⅳ		Ⅴ	
	Precision	Recall	Precision	Recall	Precision	Recall	Precision	Recall
Method 0	0.2857	0.3810	0.8636	0.7703	0.8776	0.8958	1.0000	1.0000
Method I	0.2000	0.0476	0.7667	0.9324	0.8400	0.8400	1.0000	1.0000
Method II	0.4545	0.2381	0.8072	0.9054	0.8627	0.8800	1.0000	1.0000
Method III	0.3333	0.1905	0.8161	0.9595	0.9348	0.8600	1.0000	1.0000

Compared with Method I, Methods II and III increase the number of high-quality feature sets by 142.50% and 130.37%, respectively. Method III gives the best prediction performance, improving ACC by 3.53% points and F1-score by 0.0545 over Method I. Compared with Method 0, Method III improves ACC by 4.95% points and F1-score by 0.0228. These results confirm that the proposed rock-breaking preprocessing and FPI–$\:\boldsymbol{t}$ local-maximum method better capture the rock-machine interaction and improve rock mass quality prediction.

### Engineering portability of the proposed method

#### YS project

To investigate transferability, the same modelling strategy was applied to the YS project. The optimal CatBoost model and its performance are listed in Table 11, with an ACC of 86.48%. Five-fold cross-validation indicates high stability, with an ACC of 0.8648 ± 0.0031 and a F1-score of 0.8461 ± 0.0060. Detailed results are summarized in Table 8.

**Table 11:** Parameter settings and performance of the optimal CatBoost model for the YS project.

Category	Parameter	Value
Hyperparameters	Iterations	1247
	Learning_rate	0.1878
	Depth	7
	l2_leaf_reg	12.2816
	Random_strength	0.0810
	Bagging_temperature	0.4017
Evaluation metrics	F_1_-score	0.8461
	ACC	0.8648

The same three methods (I–III) used for the YC project are then applied to the YS project to evaluate improvements in rock mass quality prediction. The results are summarized in Tables 12 and 13 .

**Table 12:** Comparison of rock mass quality prediction performance for different methods in the YS project.

Method	Number of high-quality feature sets	Prediction of surrounding rock quality			
		Training set/segments	Test set/segments	ACC/%	*F*_*1*_-score
Method I	2950	2551	399	82.71%	0.8129
Method II	3952	3553		83.46%	0.8175
Method III	4319	3920		84.96%	0.8346

**Table 13:** Detailed predictive performance metrics for the YS project.

Method	Rock mass class							
	II		III		Ⅳ		Ⅴ	
	Precision	Recall	Precision	Recall	Precision	Recall	Precision	Recall
Method I	1.0000	0.6667	0.8706	0.9212	0.7500	0.7727	0.5000	0.1000
Method II	1.0000	0.5000	0.8550	0.9544	0.7967	0.7424	0.5000	0.1000
Method III	1.0000	0.5000	0.8717	0.9585	0.8031	0.7727	0.7500	0.1500

Methods II and III increase the number of high-quality feature sets by 33.97% and 46.41%, respectively, compared with Method I. The corresponding ACC values are 82.71%, 83.46% and 84.96%. These results indicate that the proposed rock-breaking data preprocessing and FPI–t local-maximum method are also applicable to the YS project, mitigating the influence of geological conditions and tunnel diameter and exhibiting good robustness across projects.

#### HB project

**Fig. 17:**
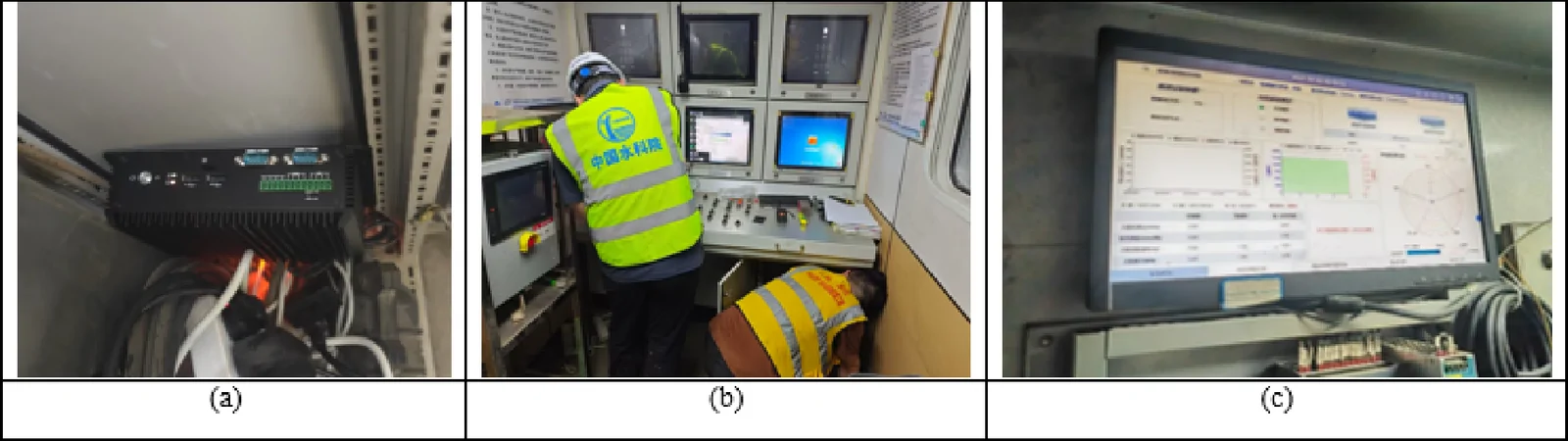
On-site deployment of the intelligent TBM tunnelling system. (**b**) Installation process, (**c**) Operator display interface.

**Fig. 18:**
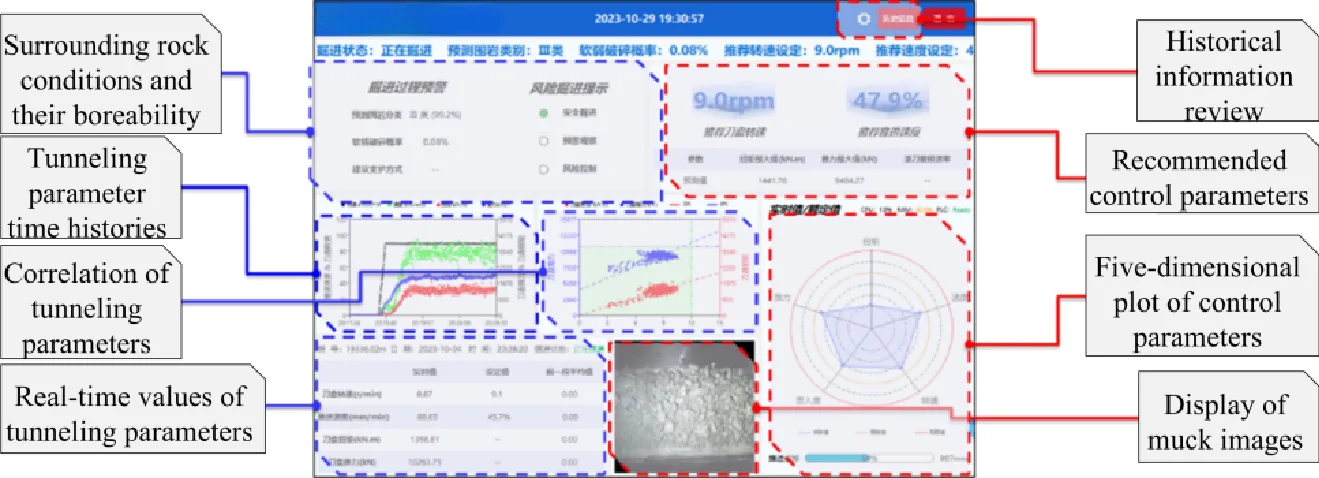
Real-time rock mass quality prediction in the TBM intelligent tunnelling system.

For the HB project, an intelligent TBM tunnelling system has been developed for TBM 9 in the Genzi branch tunnel, incorporating the characteristic parameter computation method (Method III) proposed in this study. During on-site deployment, a data acquisition unit was installed in the TBM electrical cabinet and connected to the Programmable Logic Controller(PLC) of TBM. The acquisition unit was then configured and commissioned through wiring inspection, signal verification, and synchronization tests. In addition, an industrial computer and an operator display were mounted in the control cabin to visualize real-time data and prediction results. Photographs of the data acquisition unit, the installation process, and the operator display interface are shown in Fig. 17. The system computes characteristic parameters in real time and predicts rock mass quality during tunnelling, as illustrated in Fig. 18.

**Fig. 19:**
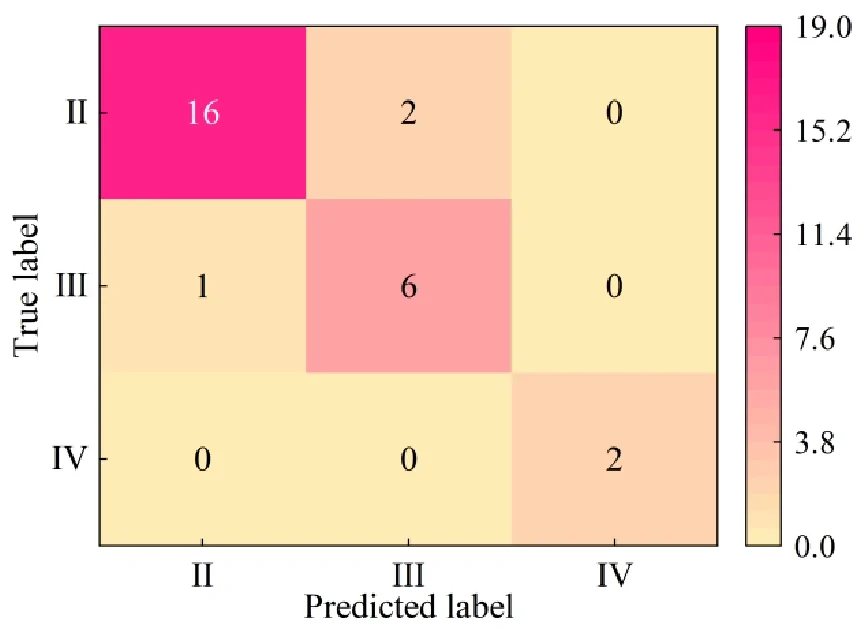
Confusion matrix of the rock mass quality prediction model for the HB project test set.

Using on-site monitoring data collected from 23 November to 24 December 2024, a total of 228 effective cycles were used for model training and prediction. Five-fold cross-validation was performed, and the results are summarized in Table 8. For the HB project, the model achieved a mean accuracy (ACC) of 0.8456 ± 0.0268 (95% CI 0.8123–0.8789) and a mean F1-score of 0.8580 ± 0.0322 (95% CI 0.8180–0.8980). Compared with YC and YS, HB exhibits relatively larger variability across folds, whereas YS is the most stable and YC lies in between. Overall, the variation across all three projects remains limited, indicating reasonably stable model performance. For reference, the confusion matrix of one representative fold (Fold 5) is shown in Fig. 19, where the overall accuracy reaches 88.89% and the F1-score is 0.8910.

**Fig. 20:**
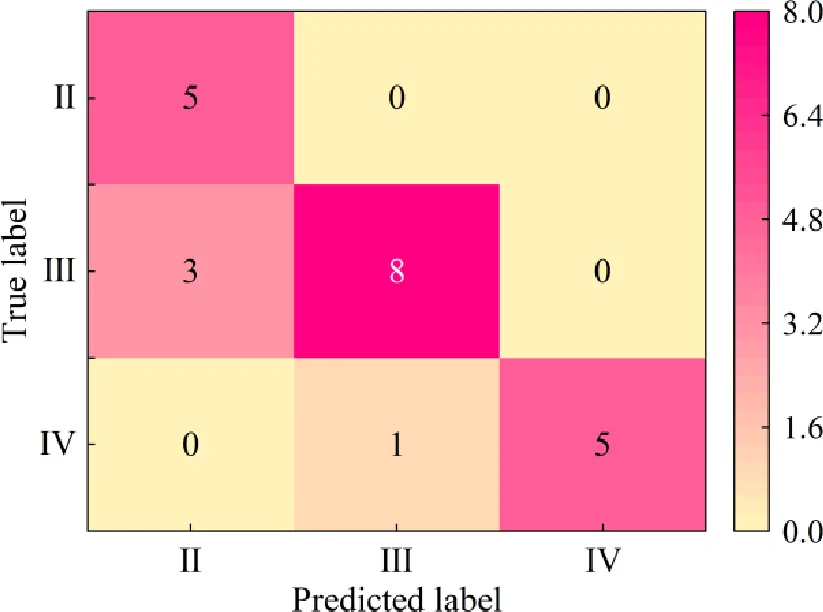
Confusion matrix for the time-based test set.

Beyond random five-fold cross-validation, we also conducted a time-based evaluation. Specifically, the most recent continuous period (e.g., 16–24 December 2024) was held out as the test set, while the remaining historical data were used for training and hyperparameter tuning; the trained model was then directly applied to the held-out period without retraining. This design aims to assess robustness under potential changes in geology and operating conditions. The resulting confusion matrix is shown in Fig. 20. Under this temporal hold-out scheme, the model achieved an accuracy (ACC) of 0.8182 and an F_1_-score of 0.8228. Relative to the random-split setting, ACC decreased by 0.0274 and the F1-score decreased by 0.0352, indicating only a modest degradation. Overall, the model remains practically usable and engineering-stable under temporal distribution shift.

**Fig. 21:**
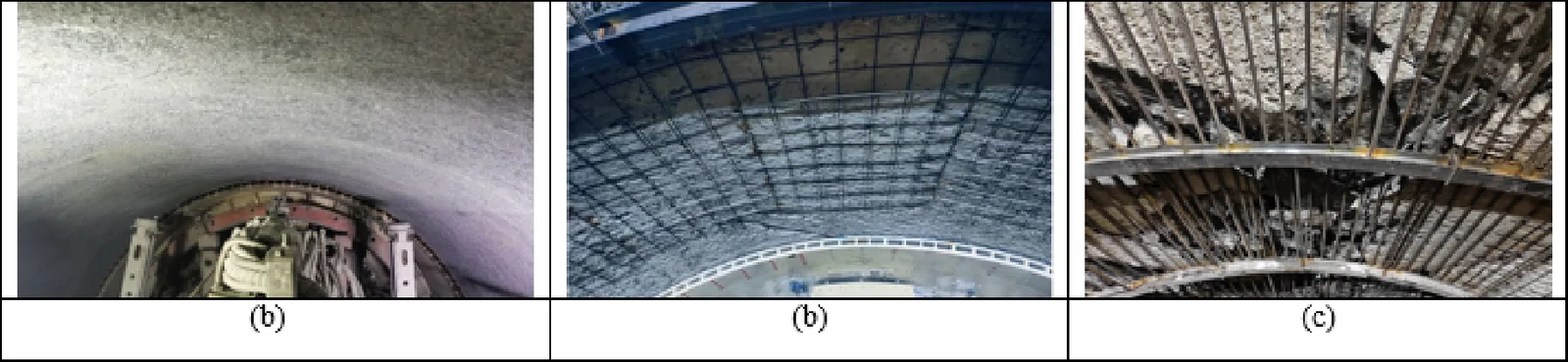
Field-observed surrounding rock conditions at different boring cycles. (**b**) Chainage 12,247.27 m, (**c**) Chainage 12,328.00 m.

The prediction results for a verifiable subset of boring cycles are further evaluated, as summarized in Table 14. Ground-truth labels for this subset were obtained from the established on-site geological rechecking and exploration record system routinely used in tunnelling projects. In Table 14, the “Observed geological conditions” are determined according to an operational criterion based on the widely adopted rock mass classification–support correlation framework in engineering practice. The in situ surrounding rock conditions at chainages 11,938.59, 12,247.27, and 12,328.00 are presented in Fig. 21. Overall, the model predictions agree well with the corresponding engineering interpretations on this subset, demonstrating reliable field follow-up performance and highlighting the practical applicability of the proposed method.

**Table 14:** Verification of prediction results for a subset of boring cycles in the test set.

Chainage (m)	Parameters			Rock mass quality and support measures		
	a	b	TPI	Predicted class	Observed geological conditions	Support method
11,938.59	80.08	129.41	7.93	II	Intact	No support
11,959.44	52.84	161.76	7.18	II	Intact	Rock-bolt support
12,175.76	38.48	195.66	7.45	II	Intact	Shotcrete support
12,313.85	37.51	159.56	5.91	III	Moderately intact	Local steel ribs
12,247.27	24.54	175.89	5.86	III	Moderately intact	Rebar mesh
12,103.97	22.77	221.46	4.45	III	Locally broken	Local steel ribs and rebar mesh
12,328.00	11.18	119.62	2.78	IV	Cavity collapse	Systematic steel ribs and rebar rows

## Conclusions

This study targets cross-project rock mass quality prediction. By filtering TBM raw data based on the effective energy conversion of disc cutter work and extracting high-efficiency rock-breaking stages considering rock fracture characteristics, a knowledge-driven real-time computation method for rock mass characteristic parameters applicable across different projects is developed. An AI-driven rock mass quality prediction model based on CatBoost is then constructed and validated using multi-project datasets. The main conclusions are as follows.

1. Rock-breaking data filtering based on disc cutter energy.

A rock-breaking data filtering method is proposed based on effective energy conversion of TBM disc cutter work. Compared with conventional data-driven cleaning methods, the proposed method achieves better performance on three real projects. The resulting CatBoost models attain prediction accuracies of 85.60% and 86.48% for the YC and YS projects, respectively, demonstrating good applicability across projects.

(2) FPI–t local-maximum method for extracting high-efficiency rock-breaking stages.

A real-time method is developed to identify the onset of the high-efficiency rock-breaking stage from the local maximum of the FPI–* t* curve. In YC and YS, this method increases the number of high-quality characteristic-parameter sets by 142.50% and 33.97%, respectively, compared with the penetration-threshold method, and improves prediction performance.

(3) Knowledge-driven cross-project computation of rock mass characteristic parameters.

Building on rock-breaking data preprocessing and high-efficiency rock-breaking stage extraction, this study proposes a knowledge-driven near-real-time method for computing rock-mass characteristic parameters. The results demonstrate good performance and engineering reproducibility in the YC and YS projects, as well as practical utility in the ongoing HB project under comparable open-type TBM conditions. Rather than claiming universal transferability, the present evidence supports promising deployment potential for similar open-type TBM projects with diameters of approximately 5–8 m. Generalization to substantially different conditions, such as shield TBM operation, ultra-deep tunnels (* >1000* m), or severe class imbalance, still requires further verification.

## Data Availability

The datasets generated and analyzed during the current study are not publicly available due to confidentiality agreements with the project owners, but are available from the corresponding author on reasonable request.

## Appendix

### Appendix A: Pseudocode for reproducing Table 2

FOR each CSV file f in folder “1.Original Data” DO.

# Rule 0: Detect encoding and read file.

enc ← chardet.detect(read_all_bytes(f)).encoding.

TRY:

df ← read_csv(f, encoding = enc).

CATCH read_error:

print(read_error).

CONTINUE.

fallback_flag ← False.

# Rule 1: Basic validity check.

IF df is empty OR n_columns(df) < 11 THEN.

fallback_flag ← True.

END IF.

# Rule 2: Displacement difference threshold.

IF fallback_flag = = False THEN.

displacement ← df[:, C10].

IF displacement[last] − displacement[1] < 300 THEN.

fallback_flag ← True.

END IF.

END IF.

IF fallback_flag = = True THEN.

# Fallback output: write a single all-zero row using the same column schema.

out_df ← one_row_zeros_with_same_columns(df.columns).

save_csv(out_df, output_dir, filename(f),

encoding = “utf-8-sig”, index = False)

CONTINUE.

END IF.

# Rule 3: Pre-filter by an upper bound on speed.

df ← df WHERE df[:, C8] < 120.

reset_index(df).

# Rule 4: Three-sigma filtering on speed.

speed ← df[:, C8].

µ ← mean(speed).

σ ← std(speed) # pandas default (ddof = 1).

df ← df WHERE (µ − 3σ) ≤ speed ≤ (µ + 3σ).

reset_index(df).

# Rule 5: Sequential rolling-min filter (window = 3) to drop rows with rolling min = 0.

candidate_cols ← [C5, C7, C8, C3] restricted to columns existing in df.

FOR col IN candidate_cols IN THIS EXACT ORDER DO.

IF df[:, col] is numeric THEN.

rmin ← rolling_min(df[:, col], window = 3, min_periods = 1)

df ← df WHERE rmin ≠ 0.

reset_index(df).

END IF.

END FOR.

# Rule 6: Remove constant groups (group size = 10) using multi-column logical AND.

colnames ← names_of_columns(df, candidate_cols).

KEEP_MASK ← True for all rows.

FOR each colname IN colnames DO.

group_id ← floor(row_index / 10).

variable_group ← (nunique(df[colname] within each group_id) > 1)

KEEP_MASK ← KEEP_MASK AND (variable_group expanded to all rows in each group).

END FOR.

df ← df WHERE KEEP_MASK is True.

# Output.

save_csv(df, output_dir, filename(f),

encoding = “utf-8-sig”, index = False)

END FOR.

### Appendix B: Histogram statistics with anomaly gating (single-parameter)

Input:

- Data table df with record index r_t in column 0.
- Target parameter column index c.

Hyperparameters:

- B: number of histogram bins (bins), default 20.
- W: smoothing window length (smoothing_window), default 5 (None means no smoothing).
- m: margin around the modal-bin threshold (margin), default 0.05.
- δj: jitter threshold (jitter_threshold), default 0.10.
- δt: trend threshold (trend_threshold), default 0.20.
- ts: previous stable index (prev_stable), optional (None if unused).

Output:

- Stable-start index t*, and its record id r_{t*}.

Steps:

1. Extract column x_t = df[t, c], keep only finite values for histogram statistics.
2. If W is not None and W > 1:

Compute centered moving average x~_t = mean(x_{t-W/2 : t + W/2})

else:

x~_t = x_t.

3. Build an equal-width histogram of {x~_t} using B bins.

Let edges be {e_0,…,e_B} and counts be {h_0,…,h_{B−1}}.

4. Find modal bin b* = argmax_b h_b, set threshold T = e_{b*+1}.

Define lower bound Tlow = T − m.

5. Sequential scan t = 0,1,2,… over the original series x_t:

- Skip if x_t is not finite.
- If x_t < = Tlow: continue.
- Jitter gating (if t > 0 and x_{t-1} != 0):

if |x_t - x_{t-1}| / |x_{t-1}| > δj: continue.

- Trend gating (if ts is not None and x_ts != 0):

if |x_t - x_ts| / |x_ts| > δt: continue.

- Return t* = t and r_{t*} = df[t, 0].

6. If no point satisfies, return t* = 0 and r_{t*} = df[0, 0].

Algorithm 2 Multi-parameter stable-start fusion (IQR + median).

Input:

- Data table df, record index in column 0.
- Set of parameters C = { (name_i, col_i) }, i = 1.M.

Hyperparameters:

- Use the same (B, W, m, δj, δt) as Algorithm 1.
- k: IQR outlier factor (outlier_iqr_factor), default 1.5.

Output:

- Final stable-start index tfinal, record id r_{tfinal}, and per-parameter details.

Steps:

1. For each parameter i in C:

(ti, ri) = Algorithm 1(df, col_i; B, W, m, δj, δt).

Collect indices T = {ti}.

2. Compute Q1, Q3 of T and IQR = Q3 - Q1.

Define keep interval [L, U] = [Q1 - k*IQR, Q3 + k*IQR].

3. Filter Tkeep = { ti in T | L < = ti < = U }.

If Tkeep is empty, set Tkeep = T.

4. Set tfinal = median(Tkeep) (cast to integer index),

r_{tfinal} = df[tfinal, 0].

5. Output tfinal, r_{tfinal}, and {ti, ri} as details.

### Appendix C: Extract cycle-level derived features (FPI/TPI) by linear fitting

Input:

- input_dir: folder containing multiple CSV files (one file per boring segment/cycle).
- output_file: path to save aggregated derived features (.csv).
- fit columns (by index):

p_up <- column 6 (penetration, used as x).

F_up <- column 7 (thrust-related term, used as y for FPI).

T_up <- column 5 (torque-related term, used as y for TPI).

TPI_col <- column 11 (existing TPI series for statistics).

FPI_col <- column 12 (existing FPI series for statistics).

- min_rows = 5, min_cols = 13.
- filter rule: keep sample if (FPI_R2 > = 0) and (TPI > = 0).

Output (per file / cycle):

- a = FPI slope.
- b = FPI intercept.
- FPI_R2 = R^2^ of FPI fitting.
- TPI = TPI slope.
- TPI_R2 = R^2^ of TPI fitting.
- TPI_mean, TPI_var (from TPI_col).
- FPI_mean, FPI_var (from FPI_col).
- Fb_fpi = mean of first 5 rows of column 7.
- Pb_fpi = mean of first 5 rows of column 6.

Procedure:

1. Initialize an empty list rows = [].
2. For each CSV file f in input_dir:
  2.1 Detect file encoding.
  2.2 Read CSV, skipping the first row; load as a numeric table.
    If read fails due to encoding, retry with UTF-8.
3. If number of rows < min_rows OR number of columns < min_cols:
  continue (skip file).
4. Extract vectors:
  x = p_up (column 6) reshaped as (− 1, 1).
  yF = F_up (column 7) reshaped as (− 1, 1).
  yT = T_up (column 5) reshaped as (− 1, 1).
5. Fit linear regression for FPI:
  Fit yF = a*x + b.
  Compute:
  a = slope, b = intercept.
  FPI_R2 = R^2^(yF, yF_pred).
6. Fit linear regression for TPI:
  Fit yT = k*x (or yT = k*x + c if desired; here keep k only).
  Compute:
  TPI = slope k.
  TPI_R2 = R^2^(yT, yT_pred).
7. Compute descriptive statistics:
  TPI_mean = mean(column 11).
  TPI_var = variance(column 11).
  FPI_mean = mean(column 12).
  FPI_var = variance(column 12).
  Fb_fpi = mean(first 5 rows of column 7).
  Pb_fpi = mean(first 5 rows of column 6).
8. Quality filtering:
  If FPI_R2 < 0 OR TPI < 0:
  continue.
9. Append a record to rows:
  {filename, a, b, FPI_R2, TPI, TPI_R2, TPI_mean, TPI_var, FPI_mean, FPI_var, Fb_fpi, Pb_fpi}
10. Convert rows to a table Results.
11. Normalize filename strings (optional): remove a fixed prefix substring.
12. Save Results to output_file with UTF-8-SIG encoding.
13. End.
